# Evaluation of Ciprofloxacin (*gyrA*, *parC* Genes) and Tetracycline (*tetB* Gene) Resistance in Nosocomial *Acinetobacter baumannii Infections*

**DOI:** 10.5812/jjm.8976

**Published:** 2014-02-01

**Authors:** Jamileh Nowroozi, Abbas Akhavan Sepahi, Lida Tahmasebinejad Kamarposhti, Roya Razavipour, Flor Mazhar

**Affiliations:** 1 Faculty of Biological Science, Islamic Azad University, North-Tehran Branch, Tehran, IR Iran

**Keywords:** *Acinetobacter baumannii*, Disinfectant, gyrA, parC, tetB

## Abstract

**Background:**

*Acinetobacter baumannii* plays an important role in some types of nosocomial infections as an opportunist microorganism which increases levels of resistance to antibacterial drugs and disinfectants.

**Objectives:**

The aim of this study was to determine the resistance and sensitivity of *A. baumannii* to different antibiotics and evaluate the minimal inhibitory concentration (MIC) for Ciprofloxacin and Tetracycline; in addition to Surfanios, Citron and Aniosyme DD1 disinfectants, and also to detect the presence of *gyrA, parC and tetB* gene bands in the isolates.

**Materials and Methods:**

In this study, 65 *A. baumannii* isolates were collected from the hospitalized patients in NIOC hospital (National Iranian Oil Company hospital) of Tehran, Iran during 2010-2011. The pattern of sensitivity to antibiotics was determined using CSLI disk diffusion and MIC methods. Furthermore, resistance of isolates to the common disinfectants (Surfanios Citron and Aniosyme DD1) was determined in different hospital wards. Presence of *gyrA*, *parC* and *tetB* gene bands was also detected by PCR method.

**Results:**

Frequency of *Acinetobacter* resistance to Amikacin, Ciprofloxacin, co-Trimoxazole, Ceftazidime and Ceftriaxone was 100% in the isolates reviewed in this study. The frequency of resistance to Gentamicin and Tetracycline were 86.1% in the isolates. The MIC of Ciprofloxacin in all (100%) of isolates was 32-64 μg/mL which showed the resistance to Ciprofloxacin In 86.1% of cases the Gentamicin and Tetracycline MIC were ≥ 16 μg/mL and in 13.9% of isolates the Gentamicin and Tetracycline MIC were 4 μg/mL, these results showed the resistance and sensitivity to the Gentamicin and Tetracycline, respectively. Additionally, all (100%) of the *A. baumannii* isolates were resistant to disinfectant concentrations, which were used with the methods recommended by manufacturers (0.5%). In 100% of the isolates *parC* and *gyrA* genes bands were detected, and *tetB* gene was also detected in 86.1% of Tetracycline resistant isolates.

**Conclusions:**

Due to the high resistance of *A. baumannii* isolates to most antibiotics in our study and also its high resistance to the common disinfectants usually used in hospitals, it seems that more attentions should be paid for applying disinfectants. Since most of the isolates were collected from tracheal and sputum samples (46%), it seems that respiratory tract is the most t prevalent site of infection among *Acinetobacter* infections. Therefore, disinfecting the respiratory tract related equipment and instruments by using proper disinfectants seems to be an appropriate way to prevent these infections.

## 1. Background

*Acinetobacter* spp. is a Gram-negative non-fermentative bacteria. These bacteria need only a few requirements for growth and they can survive on moist and dry surfaces, including the hospital environment. *Acinetobacter baumannii *plays an important role in nosocomial infections as an opportunistic pathogen in long term hospitalized patients in ICU or following long term use of antibiotics (1). *A. baumannii *is highly resistant to the most of antimicrobials agents. This resistance can be inherited or acquired through resistance genetic factors. Resistance to the antimicrobial factors in clinical samples can make the treatment of infections harder (2).

Several studies showed that the resistance of *A. baumannii *to Quinolones has a genetic basis. The most important mutations in *gyrA *and *parC *usually happen in special places called resistant determining areas. Ciprofloxacin is a wide range antibiotic for both Gram-positive and Gram-negative bacteria. This antibiotic inhibits DNA *gyrA*se, topoisomerase II and IV and production of enzymes necessary to metabolize DNA of the bacteria, thus they inhibit cell division (3).

Fluoroquinolones affects DNA through inhibition of DNA *gyrA*se (*gyrA*) and topoisomerase IV (*parC*). The main targets of fluoroquinolones in some bacteria, such as *Escherichia coli, *are DNA*gyrA*se and topoisomerase. Fluoroquinolones affect this enzyme and halt the replication and transcription (4). Quinolones have shown good activities against *Acinetobacter* spp. However, resistance to these antibiotics is rapidly increasing. Resistance to Fluoroquinolones in *Acinetobacter* species is accompanied with the mutation in the area e determining resistance to *gyrA *(QRDR) or *parC *encoding subunit A of DNA*gyrA*se and topoisomerase IV. Furthermore, mutations within the QRDRs of *gyrA *are associated with resistance to Ciprofloxacin (5). S Disinfectants are antimicrobials applied to surfaces and objects (6) which are the main measures in controlling infections and are used to prevent spreading of nosocomial infections (7).

Surgical instruments, such as needles, syringes, instruments used in association with blood vessels, urinary catheters, arthroscopies and laparoscopes should be sterile, due to the possibility of transmitting infections. Level of activity of biocides depends on several factors. Some of these factors are related to the type of biocide and others relate to the microorganisms (8).

## 2. Objectives

The purpose of this study was to determine the resistance and susceptibility of *A. baumannii *to various antibiotics, Surfanios and Aniosyme DD1, the MIC of Ciprofloxacin and Tetracycline and to detect the presence of *gyrA*, *parC *and *tetB *gene bands in isolated bacteria.

## 3. Materials and Methods

**Figure 1.**
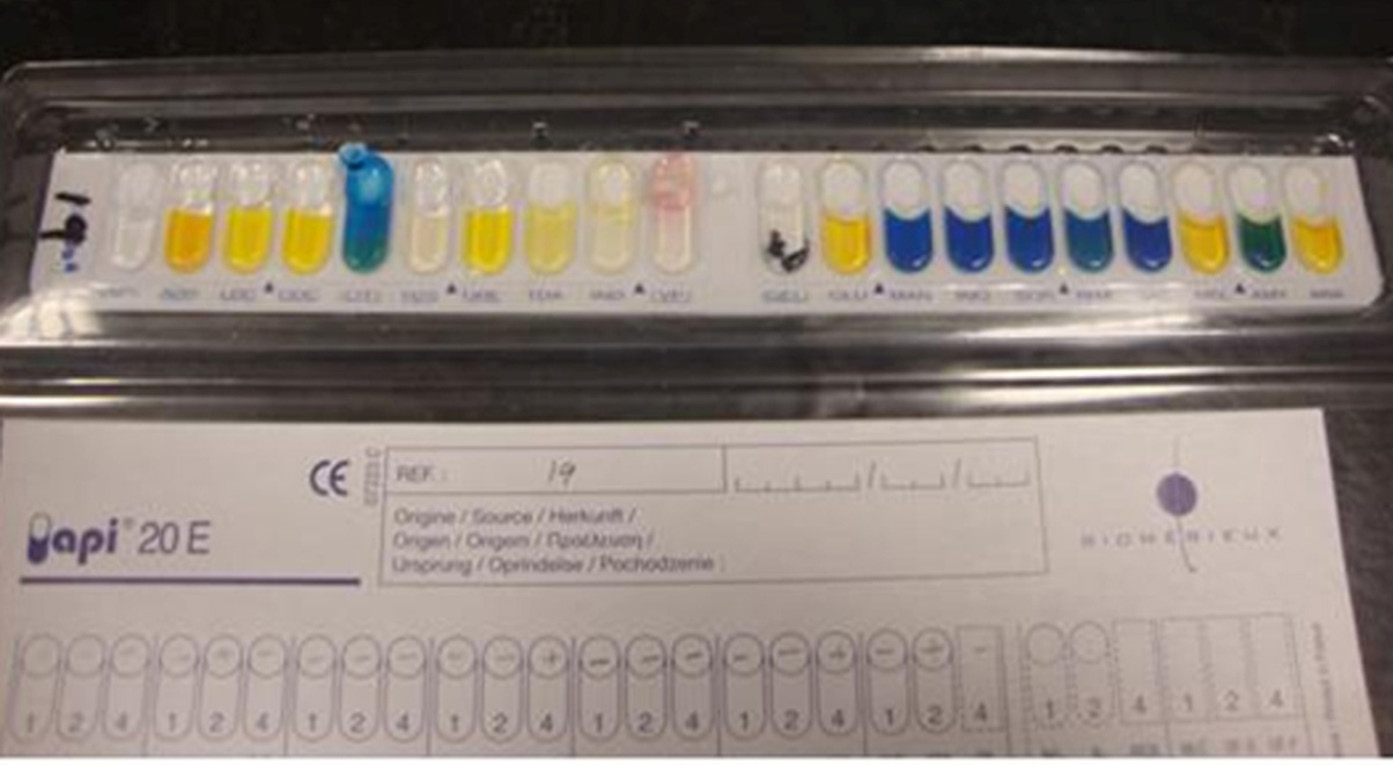
The Results of Biochemical Tests for *Acinetobacter baumannii* With API Kit

**Figure 2.**
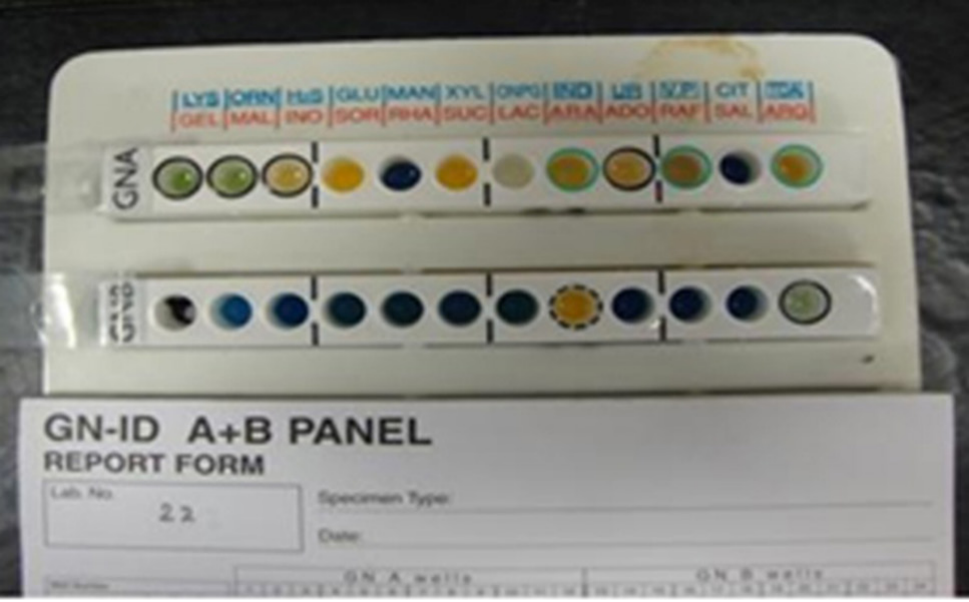
The Result of Biochemical Tests for *Acinetobacter baumannii* With Microgen kit

This research was conducted between years 2010 and 2011 in Mahmoodieh laboratory of Islamic Azad University, North Branch to fulfill the requirements for a MSc thesis. 2500 samples were collected from December 2010 to August 2011 from the respiratory secretions, urine, wounds, sputum and blood samples of patients hospitalized in one of Tehran’s hospitals. In order to identify *Acinetobacter*, biochemical tests (Oxidase, Catalase, OF Glucose, TSI, Indol ]SIM[, motility ]SIM[, Simon Citrate, Growth on MacConkey (Merck Co., Germany) and growth in 42°C) were performed and also to confirm the API 20E (Biomerieux Co., France) diagnostic kits and MICROGEN GNA+B (Microgen Bioproducts Co., UK) were used (Figures 1, 2). 

Then these bacteria were stored in media containing glycerol at -20°C. Screening for different antibiotic resistance (Ciprofloxacin, Ceftazidime, Ceftriaxone, Tetracycline, Gentamicin, Amikacin and co-Trimoxazole) was performed using disk diffusion test according to Clinical and Laboratory Standards Institute (CLSI) (PadtanTeb Co., Iran). Disks were placed on Mueller-Hinton agar (Merck co.Germany) and incubated for 24h at 35°C. The results were compared to that of the CLSI. The minimum inhibitory concentration (MIC) of *A. baumannii *for Ciprofloxacin and Tetracycline were performed according to macro and micro dilution broth methods based on CLSI guidelines. The MIC assay is a technique used to determine the lowest concentration of a particular antibiotic needed to kill bacteria. Antibiotic powders were dissolved in sterile deionized water or an appropriate solvent according to the manufacturer’s recommendations. Test concentrations for antibiotics were 256 g/mL, 128 g/mL, 64 g/mL, 32 g/ml, 16 g/mL, 8 g/mL, 4 g/mL, 2 g/mL, 1 g/mL 0.5 g/mL and 0.25 g/mL. Each well of a microtiter plate contained a total volume of 100 mL concentrated antibiotic and Mueller-Hinton medium with the bacterial inoculums and TTC (triphenyletetrazolium chloride). Micro plates were incubated at 35°C for 18h to 24h. The lowest concentration of antibiotic without visible bacterial growth was defined as the MIC (9).

Resistance and sensitivity of isolated bacteria to the Surfanis disinfectant (containing didecyl- dimethyl chloride) and 0.2, 0.5 and 1% Aniosyme DD1 (containing polyhexamethylene hydrochloride) were assessed by spotting and disk diffusion methods.

### 3.1. Extraction of DNA and Performing PCR

DNA of isolates was extracted according to manufacturer’s protocol using (MBST) and PCR kits. Primers were provided by Rubin TebGostar Co and PCR kit was provided by Metabion Co. For PCR amplification, 1 mL of the extracted DNA was transferred to 24 μL of the PCR amplification mix consisting of: 2.5 μL of buffer, 1 μL of dNTP, 18 μL of sterile deionised water, 1 μL of each primers ( forward and reverse) and 0.5 μL of Tag polymerase. For *gyrA*, the following parameters were used: an initial template denaturation at 95°C for 1 min; 36 cycles consisting of 30s of denaturation at 95°C, 30s of annealing at 53°C and 2 min of extension at 72°C; and a final extension at 72°C for 10 min. For *parC*: an initial template denaturation at 95°C for 2 min; 36 cycles consisting of 1 min of denaturation at 95°C, 1 min of annealing at 60°C and 2 min of extension at 72°C; and a final extension at 72°C for 10 min and for *tetB*an initial template denaturation at 95°C for 5 min; 25 cycles consisting of 30s of denaturation at 95°C, 30s of annealing at 57°C and 20s of extension at 72°C; and a final extension at 72°C for 7 min.

To perform PCR, nucleotide sequences of the used primers for *gyrA*, *parC *and *tetB* were as follows:

*gyrA*F: 5′AAATCTGCCCGTGTCGTTGGT3′ 16
*gyrA*R: 5′GCCATACCTACGGCGATACC3′
*parC*F: 5′AAAAATCAGCGCGTACAGTG3′ 16
*parC*R: 5′CGAGAGTTTGGCTTCGGTAT3′
*tetB F*: 5′TACGTGAATTTATTGCTTCGG3′ 17
*tetB R*: 5′ATACAGCATCCAAAGCGCAC3′

For electrophoresis of PCR products, 1% agarose gel was used. Afterwards the gel was stained with ethidium bromide and imaged.

## 4. Results

Among collected samples, 65 *A. baumannii *(2.6% of total samples) were isolated, of which 36.9% were from tracheal, 27.6% from wound, 18.4% from urine, 9.2% from sputum and 7.6% from blood samples. These bacteria grew well in blood agar, MacConkey and BHI agar. *A. baumannii is *catalase positive and oxidase negative which differentiates it from Pseudomonas. These bacteria were ALK/ALK positive in TSI medium and citrate while urea and Indol were negative. SIM medium was used for Indol test and Urease medium for urea test also gelatin test was negative. It mentioned that these bacteria don’t have Gelatinase so couldn’t liquefy gelatin medium. Production of acid in OF Glucose was positive. In order to differentiate *A. baumannii* from *A. calcoaceticus*, the plate was kept in 42 ˚C.

All cases (100%) had negative inhibition zones for Ciprofloxacin, Amikacin, co-Trimoxazole, Ceftriaxone and Ceftazidime and most cases (86.1%) for tetracycline and Gentamicin. Inhibition zone for tetracycline and gentamicin was observed in 13.9% of isolates based on the CLSI table. Resistance to disinfectants Surfanios and DD1 with %0.5 concentration of (according to the manufacturer’s instruction) was observed in (all) 100% of bacteria, but all (100%) of bacteria were sensitive to double concentrations of these disinfectants. 

**Figure 3.**
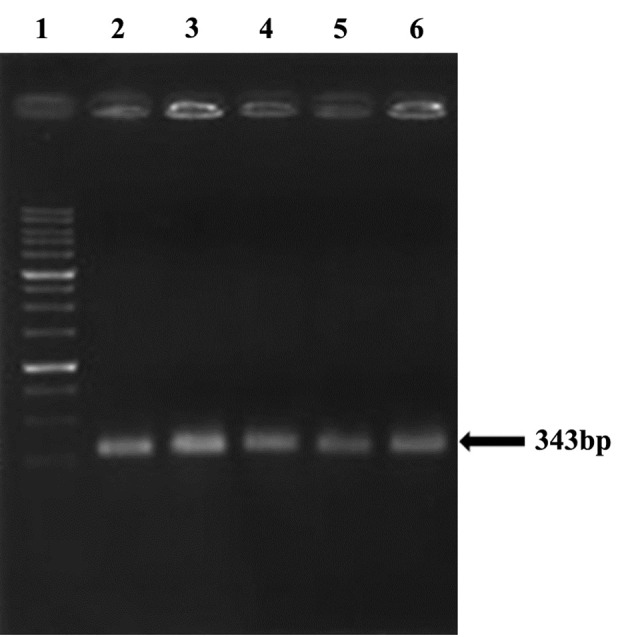
gyr A

**Figure 4.**
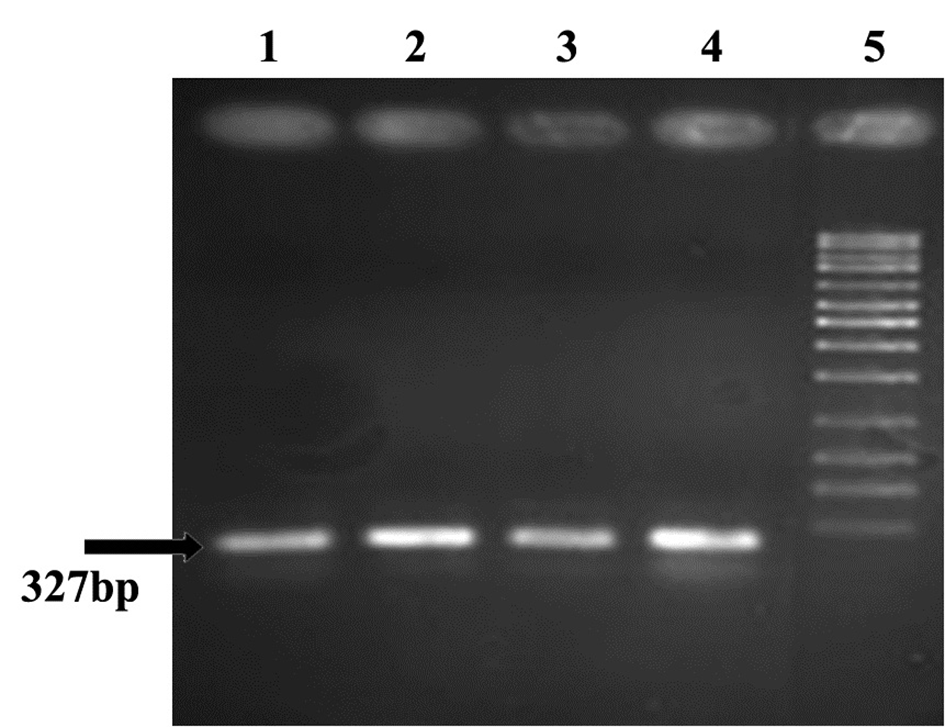
parC

**Figure 5.**
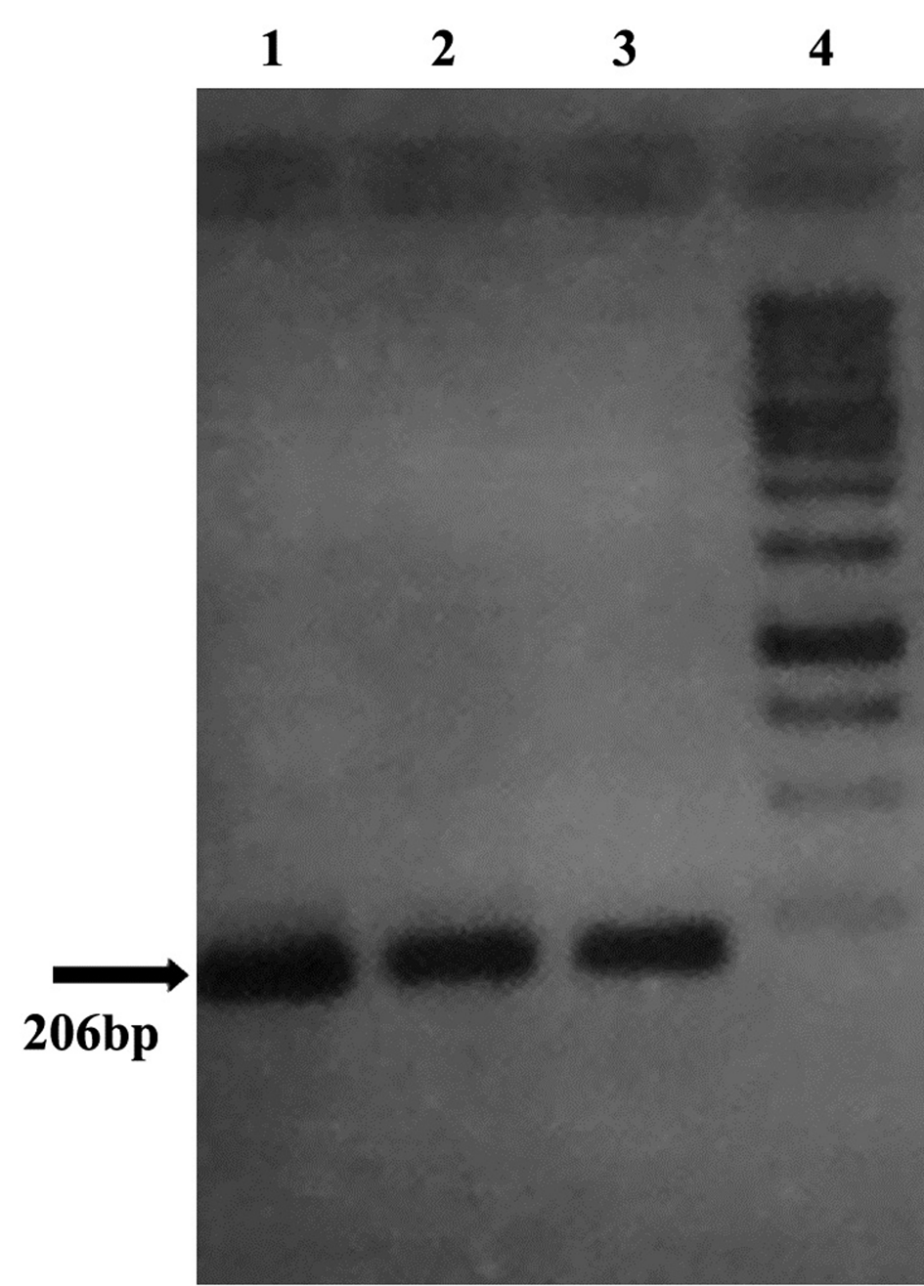
tet B

All (100%) *A. baumannii *were resistant to Ciprofloxacin with MIC of 32-64 μg/mL, and were resistant to tetracycline and Gentamicin in 86.1% of cases with MIC of ≥ 16 μg/mL and also sensitive in 13.9% of isolates with the MIC of 0-4μg/mL. Our results showed that all (100%) of isolates were resistant to Ciprofloxacin, Ceftazidime, Amikacin, Ceftriaxone, and co-Trimoxazole, and also 86.1% of isolates were resistant to tetracycline and Gentamicin. In all isolates (100%) *gyrA *and *parC* gene bands were detected (Figures 3, 4). *TetB *gene band was also found in 86.1% of isolates (Figure 5) which were resistant to tetracycline but not in sensitive strains.

## 5. Discussion

*A. baumannii *is one of the most important bacteria in nosocomial infections. When the consumption rate of an antibiotic is high, antibiotic resistant microorganisms emerge due to the cessation of growth of sensitive strains, and the increase of growth in resistant strains. Resistance rate varies from one antibiotic to the other and from one microorganism to another (10). Nowadays, due to wide application of antibiotics in treatment of diseases, resistant strains appear more often (11). Resistance of single bacteria to multiple antibiotics causes severe problems in treatment of diseases.

Resistance of *A. baumannii *to multiple antibiotics is not a new phenomenon: this bacteria, is inherently resistant to many antibiotics due to its ubiquitous existence, and its resistance rate is also increasing (12). Results of our study showed that *A. baumannii *had high resistance rates to most antibiotics. *A. baumannii *was resistant to Amikacin, Ciprofloxacin, co-Trimoxazole, Ceftazidime and Ceftriaxone in 100% of isolates and the frequency of resistance to gentamicin and tetracycline were 86.1%. In all (100%) of isolated bacteria, resistance to at least three antibiotics was observed which was identified as multiple drug resistant (MDR).

MDR in *A. baumannii *leads to problems in treating patients with infections caused by this bacteria. In studies of Hujer et al antibiotic sensitivity tests were done according to the CLSI criteria. The results showed that eighty nine percent of the collected *Acinetobacter *species were resistant to at least three groups of antibiotics. In fact, they showed MDR. They reported that more than 90% of samples were resistant to Ciprofloxacin and 81% resistant to Amikacin or Tobramycin (13). In another study, Srinivasan et al. have shown that *A. baumannii *was resistant to the following antibiotics: Imipenem, Ceftazidime, Amikacin, Streptomycin, Gentamicin, Kanamycin, Tetracycline, Ciprofloxacin and Nalidixic acid. Susceptibility of *Acinetobacter* to these antibiotics was determined by broth dilution method.

Seventy nine and a half percent of isolated *A. baumannii *were resistance to Ceftazidime, Amyimipenem, Amikacin, Kanamycin, Gentamicin, Streptomycin, Tetracycline, Ciprofloxacin and Nalidixic acid, i.e. They found out that 79.5% of isolated *Acinetobacter* species were MDR (14). In another study by Adams-Haduch et al, the rate of complete resistance to antibiotics were as follows: 95.9% showed resistance to Ciprofloxacin, 87.7% To Ceftazidime, 79.6% To Cefepime, 40.8% To Ampicillin sulbactam, 18.4% to Imipenem 22.4% To Meropenem, 36.7% To Amikacin, 61.2% to Tobramycin, 77.6% to Gentamicin and 79.6% to Tetracycline. Totally, 16.3 % were resistant to six groups of experimented antibiotics (15).

In the study of Vila et al. *Acinetobacter* species which were resistant to Ciprofloxacin with MIC ≥4 showed a mutation of *gyrA* gene in PCR analysis (16). In the study of Jordi Vila *gyrA *gene mutations were detected in strains with MIC≥4 μg/mL, and *parC* gene mutations were detected in strains with MIC≥32 μg/mL (17). In the study of Seward RJ and Towner KJ *Acinetobacter* species which were resistant to ciprofloxacin (MIC ≥ 4) had the *gyrA *gene mutation and one of resistant bacteria with MIC≥ 64 μg/mL had both the *parC *and *gyrA* gene mutations (18). In a study by Hujer et al, 88% e of *A. baumannii *were resistant to Ciprofloxacin, but *parC* gene mutation was detected only in one isolate (13). The mentioned studies have shown that mutations in both *gyrA *and *parC*are genes are required to develop high level of resistance to Ciprofloxacin and this is probably due to effect of these mutations on the permeability of *A. baumannii *to quinolones (19).

In the study reported by Lee et al *A. baumannii *was recognized as an opportunistic pathogen in nosocomial infections and that the worldwide resistance to Fluoroquinolones is rapidly increasing. The mechanism of resistance to Fluoroquinolone is mainly associated with quinolone resistance determining regions (QRDR) from *gyrA locus *related to DNA *gyrA*se and *parC *genes encoding topoisomerase IV. *GyrA *and *parC mutations *were observed in bacteria resistant to Ciprofloxacin (20). In the present study, all strains had MIC≥ 32 μg/mL; *gyrA *gene mutation was detected in MIC ≥4μg/mL and *parC* band in MIC≥ 32 μg/mL. Therefore, all isolates had *gyrA *and *parC *gene mutations and this accounts for the high level of resistance of *A. baumannii *to Ciprofloxacin.

Because of the outbreak of *A. baumannii *in our hospital, especially intensive care unit (ICU), efficacy of disinfectants such as Surfanios (which were used for the surfaces) and Aniosyme DD1 (which were used for the medical instruments) were also evaluated. Results showed that all (100%) of bacteria were resistant to disinfectants at the recommended concentrations by company (0.5%), but they were susceptible to these detergents when used in double concentrations. Since most of the samples in this study were taken from tracheal secretions and sputum, probably the main sources of infections were equipments used in the respiratory system. Therefore, disinfection of respiratory system related equipments is one important way of preventing infections. It was recognized in this study that these disinfectants were applied for two years, resistance to the applied concentrations was developed and consequently stably remained in the environment which resulted in outbreaks in different units of the hospital. Also, it is probable that the high resistance of *A. baumannii *to different antibiotics is associated with its resistance to disinfectants.

Based on the results of the present study, the most important measure to control high level resistance in hospitalized patients in special care unit is to monitor and the use of broad spectrum antibiotics and prescription of antibiotics with high dosage. Thus, as *A. baumannii *has become resistant to many antibiotics, use of the above mentioned antibiotics is not recommended. Also, as the many studies have shown the outbreak of these bacteria in Iran and many other parts of the world, installation of preventive measures is recommended. Although, it seems that disinfectants are the primary defense line to prevent spreading of infections, it is believed that high usage of these antimicrobial substances may cause selective pressure and results in resistance of microorganisms to disinfectants. Besides, several reports have shown the possibility of resistance to antibiotics following the inappropriate and overuse of biocides. Therefore, the appropriate use of disinfectants and changing them every six to twelve month is recommended.

## References

[1] Fagon JY, Chastre J, Domart Y, Trouillet JL, Gibert C (1996). Mortality due to ventilator-associated pneumonia or colonization with Pseudomonas or Acinetobacter species: assessment by quantitative culture of samples obtained by a protected specimen brush.. Clin Infect Dis..

[2] Bou G, Oliver A, Martinez-Beltran J (2000). OXA-24, a novel class D beta-lactamase with carbapenemase activity in an Acinetobacter baumannii clinical strain.. Antimicrob Agents Chemother..

[3] Singh B, Mitchison DA (1954). Bactericidal activity of streptomycin and isoniazid against tubercle bacilli.. Br Med J..

[4] Malek Nejad P, Arjmand M, Sotoudenia AH, Mansourirad AR (2006). Jautz summary and medical microbiology tests..

[5] Ling TK, Ying CM, Lee CC, Liu ZK (2005). Comparison of antimicrobial resistance of Acinetobacter baumannii clinical isolates from Shanghai and Hong Kong.. Med Princ Pract..

[6] McDonnell G, Russell AD (1999). Antiseptics and disinfectants: activity, action, and resistance.. Clin Microbiol Rev..

[7] Sheldon AT (2005). Antiseptic “resistance”: real or perceived threat?. Clin infect Dis..

[8] (2009.). Http://ec.europa.eu/health/opinions/en/biocidesantibiotic-resistance/l-3/index.htm.

[9] Performance Standards for Antimicrobial Susceptibility testing (2007). Clinical and laboratory standards institute, 19th informational supplement (M100-S19)..

[10] Fux CA, Stoodley P, Hall-Stoodley L, Costerton JW (2003). Bacterial biofilms: a diagnostic and therapeutic challenge.. Expert Rev Anti Infect Ther..

[11] Arias CA, Murray BE (2009). Antibiotic-resistant bugs in the 21st century--a clinical super-challenge.. N Engl J Med..

[12] Weaver RE, Actis LA (1994). Identification of Acinetobacter species.. J Clin Microbiol..

[13] Hujer KM, Hujer AM, Hulten EA, Bajaksouzian S, Adams JM, Donskey CJ (2006). Analysis of antibiotic resistance genes in multidrug-resistant Acinetobacter sp. isolates from military and civilian patients treated at the Walter Reed Army Medical Center.. Antimicrob Agents Chemother..

[14] Srinivasan VB, Rajamohan G, Pancholi P, Stevenson K, Tadesse D, Patchanee P (2009). Genetic relatedness and molecular characterization of multidrug resistant Acinetobacter baumannii isolated in central Ohio, USA.. Ann Clin Microbiol Antimicrob..

[15] Adams-Haduch JM, Paterson DL, Sidjabat HE, Pasculle AW, Potoski BA, Muto CA (2008). Genetic basis of multidrug resistance in Acinetobacter baumannii clinical isolates at a tertiary medical center in Pennsylvania.. Antimicrob Agents Chemother..

[16] Vila J, Ruiz J, Goni P, Marcos A, Jimenez de Anta T (1995). Mutation in the gyrA gene of quinolone-resistant clinical isolates of Acinetobacter baumannii.. Antimicrob Agents Chemother..

[17] Vila J, Ruiz J, Goni P, Jimenez de Anta T (1997). Quinolone-resistance mutations in the topoisomerase IV parC gene of Acinetobacter baumannii.. J Antimicrob Chemother..

[18] Seward RJ, Towner KJ (1998). Molecular epidemiology of quinolone resistance inAcinetobacterspp.. Clin Microbiol Infect..

[19] Hamouda A, Amyes SG (2004). Novel gyrA and parC point mutations in two strains of Acinetobacter baumannii resistant to ciprofloxacin.. J Antimicrob Chemother..

[20] Lee JK (2007). Genetic analysis of fluoroquinolone-resistant Acinetobacter baumannii isolates from Korea.. European Congress of Clinica..

